# Exploring through the use of physiological and isotopic techniques the potential of a PGPR-based biofertilizer to improve nitrogen fertilization practices efficiency in strawberry cultivation

**DOI:** 10.3389/fpls.2023.1243509

**Published:** 2023-09-15

**Authors:** Jesús V. García-López, Susana Redondo-Gómez, Noris J. Flores-Duarte, María Zunzunegui, Ignacio D. Rodríguez-Llorente, Eloísa Pajuelo, Enrique Mateos-Naranjo

**Affiliations:** ^1^ Servicio General de Invernadero, Centro de Investigación, Tecnología e Innovación de la Universidad de Sevilla (CITIUS), Seville, Spain; ^2^ Departamento de Biología Vegetal y Ecología, Facultad de Biología, Universidad de Sevilla, Seville, Spain; ^3^ Departamento de Microbiología y Parasitología, Facultad de Farmacia, Universidad de Sevilla, Seville, Spain

**Keywords:** chlorophyll fluorescence, intensive farming practices, gas exchange, PGPR-based biofertilizers, nitrogen, strawberry

## Abstract

The use of microorganisms as a biofertilizer in strawberry has focused mainly on pathogen biocontrol, which has led to the underestimation of the potential of microorganisms for the improvement of nutritional efficiency in this crop. A study was established to investigate the impact of a plant growth-promoting rhizobacteria (PGPR) based biofertilizer integrated by self-compatible stress tolerant strains with multiple PGP properties, including atmospheric nitrogen fixation, on strawberry (Fragaria × ananassa cv. Rociera) tolerance to N deficiency in terms of growth and physiological performance. After 40 days of nitrogen fertilization shortage, inoculated plants were able to maintain root development and fertility structures (i.e. fruits and flowers) at a level similar to plants properly fertilized. In addition, inoculation lessened the negative impact of nitrogen deficiency on leaves’ dry weight and relative water content. This effect was mediated by a higher root/shoot ratio, which would have allowed them to explore larger volumes of soil for the acquisition of water. Moreover, inoculation was able to buffer up to 50% of the reduction in carbon assimilation capacity, due to its positive effect on the diffusion efficiency of CO_2_ and the biochemical capacity of photosynthesis, as well as on the activity of photosystem II light harvesting. Furthermore, the higher leaf C/N ratio and the maintained δ^15^N values close to control plants were related to positive bacterial effects at the level of the plant nutritional balance. Despite these positive effects, the application of the bacterial inoculum was unable to completely counteract the restriction of fertilization, being necessary to apply a certain amount of synthetic fertilizer for the strawberry nutrition. However, according to our results, the complementary effect of this PGPR-based biofertilizer could provide a higher efficiency in environmental and economic yields on this crop.

## 1 Introduction

Crop intensification, which increases crop yields to satisfy the increasing demand for resources of a continuously growing worldwide population by using a large amount of inputs such as water, biocides and fertilizers, has been associated with important environmental problems (Foley et al., 2011; Tilman et al., 2011; Davis et al., 2016). Among the primary detrimental effects on the environment are land use change, greenhouse gas emissions due to agricultural mechanization, the steady depletion of global water resources (Falkenmark and Rockström, 2004; Mekonnen and Hoekstra, 2014) and their contamination due to excessive farm use of nitrogen fertilizers.

Nitrogen contamination by the fertilization process of intensive farming practices has been recognized as one of the main factors that release several undesired effects, such as contamination of water bodies and/or lost biodiversity that jeopardize ecosystem conservation (Barros et al., 2012; Martinez-Dalmau et al., 2021). This problem is even more serious if one considers that current nitrogen fertilization practices are highly inefficient. In fact, half of the supplied N fertilizer has been described to be lost and therefore not used by crops (Billen et al., 2013; Marinov and Marinov, 2014) and with the consequent economic impact and environmental risk.

The European Union Farm-to-Fork Strategy envisages as one of its main objectives to limit nutrient losses by a minimum 50% by 2030 by reducing fertilizer application in agricultural practices by at least 20% (EUR-Lex - 52019DC0640). Significant economic and environmental costs associated with excessive fertilizer application and reduced growth and economic return resulting from nutrient deficiencies are strong incentives to optimize fertilizer application by striking a balance (Morales and Warren, 2012).

Intensive work has been done to optimize N fertilization practices in intensive crops. Among them are included: (i) development of breeding programs to obtain more efficient crops in nitrogen absorption (Hartmann et al., 2015; Xu et al., 2023); (ii) the use of innovative stabilized fertilizers (Yadav et al., 2016; Xu et al., 2023) (iii); the implementation of more precise fertilizer application techniques (Xu et al., 2023); and (iv) carry out a fertilizer application adjusted to the knowledge of the real needs of the crop and nutritional soil characteristics (Lhada et al., 2005; Arregui and Quemada, 2006). Although there have been advances, intensive crop cultivation still uses a large amount of agrochemicals, with the consequent economic costs for producers and the aforementioned ecological risk. For this reason, one of the main goals of intensive farming is to boost research aimed at optimizing the fertilization process, minimizing the use of chemical fertilizers and thereby reducing production costs, and preventing those risks caused by the leaching of these compounds to surface and underground waters.

The microbiota naturally present in the soil or supplied in the form of biofertilizers appears as a promising tool to maintain intensive agricultural production, by improving soil conditions (Ruzzi and Aroca, 2015; Zaidi et al., 2015). In this way, some beneficial bacteria, collectively known as plant growth-promoting rhizobacteria (PGPR), have a positive impact on plants by contributing to the growth of the root system or by strengthening plant tolerance to a number of environmental stresses, including nutritional-induced stress (Glick, 2012). These positive effects are mediated through different direct positive impacts of bacterial properties (Adesemoye et al., 2009), such as atmospheric nitrogen fixation, siderophores production with improved iron uptake, phosphorous solubilization, and biofilm formation, which improves bacterial adhesion to root tissue, facilitating plant nutrient uptake (Glick, 2012; Spolaor et al., 2016; Valle-Romero et al., 2023). Furthermore, other bacterial properties such as auxin production play a prominent role in root development and therefore in improving plant capacity to explore soil volume and absorb nutrients (Remans et al., 2008; Ljung, 2013). Despite the potential of using PGPR to enhance the productivity of different intensive crops (Glick, 2012; Zaidi et al., 2015), there are a small number of commercialized biofertilizers consisting of PGPR consortiums (Etesami and Maheshwari, 2018), a fact that calls for the need to design PGPR-based biofertilizer to reduce the dependence of intensive agriculture on the application of large amounts of chemical input.

Strawberry (*Fragaria* × *ananassa* cv. Rociera) is a useful plant to investigate the effect of PGPR-based biofertilizers on plant tolerance and nutritional balance under the scenario of chemical nitrogen fertilization shortage, as this is a world-important crop in terms of yield and economical cost effectiveness (FAO, 2023), especially under the integrated production system within plastic greenhouses using a drip irrigation and fertilization system (Balasooriya et al., 2018). In addition, its intensive farming character makes it highly dependent on a great amount of input application, such as chemical fertilizers (Martínez-Ferri et al., 2016). Thus, fertirrigation takes place throughout the production cycle with high daily frequency, as most crops in the southwest of Spain are characterized by low retention capacity of water and fertilizers (Ariza et al., 2012). These aspects have led to environmental issues such as pollution of water bodies by residues of chemical fertilizers (Bottoms et al., 2013; Lozano et al., 2016). This is particularly important in areas at increased risk of environmental degradation, such as Doñana National Park, a UNESCO World Heritage Site where many strawberry crops are located in Spain.

Taking these aspects into account, we hypothesize that a PGPR-based biofertilizer designed with self-compatible stress tolerant strains isolated from halophytic plant species with multiple plant growth-promoting properties (PGP), including atmospheric nitrogen fixation, could contribute to improve *Fragaria x ananassa* cv. Rociera tolerance to stress induced by nitrogen fertilization shortages, allowing strawberry producers to reduce the chemical nitrogen supply in a large proportion. Thus, we address the following concrete questions: (i) would biofertilizer application have a beneficial impact on plant performance under nitrogen fertilization shortage induced stress? And (ii) would this response be mediated by bacterial inoculation modulation effect on the different degrees of specific plant tolerance mechanisms activation? The answers to these questions are of great scientific interest, since the use of microorganisms as biofertilizers to improve the strawberry crop has focused mainly on pathogen biocontrol, with very few and recent studies analyzing its potential for improving strawberry tolerance to environmental stress, including nitrogen deficiency. This circumstance has led to an underestimation of the potential of microorganisms as useful biotools to improve nutritional efficiency in intensive crops such as strawberries. Furthermore, it is necessary to understand the tolerance mechanisms triggered by bacterial inoculation to determine the real potential of this biotool.

## 2 Materials and methods

### 2.1 Experimental design and PGPR-based biofertilizer characteristics

*Fragaria* × *ananassa* cv. Rociera plants were supplied by the company Agro GM SL and left in a chamber at 5°C until transplanted into 3.5 L plastic pots filled with sterilized vermiculite substrate (substrate composition: SiO2 34–43%, Al2O3 7–15%, Fe2O3 5–13%, MgO 20–28%, CaO 0.2–1%, TiO2 0.01-0.1% and grain size of 0.5-3 mm; Massó Garden SA) and placed in a greenhouse (37°240 N, 6°00 W; SW Spain) under controlled environmental conditions, 25 ± 4° C, 40-50% RH, subjected to a day/night regime of 16 h of light (maximum photosynthetic photon flux density (PPFD) incident on leaves of 1000 μmol m^-2^ s^-1^) and 8 h of darkness. Then plants were divided into three treatments of ten individuals per group and arranged in a randomized plot. The established treatment groups were: i) 100% nitrogenous chemical fertilization, where the buffered nodulation nutritional medium [BNM; Ehrhardt et al. (1992)] was supplemented with 5 mmol L^-1^ KNO_3_, starting from a stock solution at a concentration of KNO_3_ 1 mol L^-1^, control treatment; ii) fertilization with BNM medium without N addition and with the application of PGPR inoculum, BNM In treatment; and iii) fertilization with BNM medium without N addition and inoculation, BNM N-In treatment. During experiment, fertilization and irrigation were developed manually twice a week with a separation of 3 days (applying 100 mL per plant of a specific BNM solution). Bacterial inoculation was carried out at the beginning of the experiment and on days 15 and 30 of the experiment, applying 100 mL of a previously designed bacterial inoculum.

The bacterial inoculum used in this study was composed by five self-compatible bacterial strains with high multi-stress resistance and a variety of complementary plant growth-promoting properties (PGP), characterized and used in several of our previous studies (Andrades-Moreno et al., 2014; Mesa et al., 2015; Mesa-Marín et al., 2020; Redondo-Gómez et al., 2022; Valle-Romero et al., 2023; see **Table 1** for details of PGP properties). For the technical details of inoculum preparation see Navarro-Torre et al. (2016) and Flores-Duarte et al. (2022).

**Table 1:** Plant growth-promoting (PGP) properties of biofertilizer bacterial strains.

PGP	SDT3	HPJ40	SMT38	SRT15	S110
Phosphate solubilizing	+	+	–	+	–
Siderophores production	+	+	+	–	+
IAA production		–	–	+	+
Fixation of nitrogen	–	+	–	+	+
Biofilm formation	–	+	+	+	+
ACC deaminase activity	–	–	+	–	+

IAA, indole-3-acetic acid; SDT3, Pseudomonas sp; HPJ40, B. zhangzhouensi; SMT38, B. velezensis; SRT15, P. oxydans; S110, V. paradoxus; +, positive; -, negative.

### 2.2 Analysis of plant growth and physiological traits

Fresh weight and dry mass plant fractions were recorded after 40 days of the onset of treatments (n = 10 replicates per treatment; for details, see Redondo-Gómez et al., 2021). Just before the end of the experiment, five net CO_2_ assimilation rate versus calculated intercellular CO_2_ concentration curves (A_N_/C_i_) with 12 different ambient CO_2_ concentration (C_a_) values per nitrogen fertilization treatment were developed in fully developed leaves, using an infrared gas analyzer (LI-6800-01, LICOR Inc., Lincoln, NE, USA; see Valle-Romero et al., 2023 for the characteristics of the A_N_/C_i_ curves). And through parameterization of these curves, the mesophyll conductance (g_m_) and the maximum carboxylation rate allowed by carboxylase/oxygenase of ribulose-1,5-biphospate (RuBP) (*V*_c,max_) were obtained following the recommendations of Flexas et al. (2009, 2013), Pons et al. (2009) and Long and Bernacchi (2003). These parameters were used together with the recorded net CO_2_ assimilation rate (A_N_), stomatal conductance (g_s_), intercellular CO_2_ concentration (C_i_) and dark respiration rate (R_d_) measurements to determine the absolute limits of photosynthetic activity due to nitrogen fertilization limitation according to the Grassi and Magnani (2005) approach.

On the other hand, photochemical traits of plants were obtained in fully developed leaves from each experimental treatment (n = 10 replicates per treatment) coupled to a multiphase flash fluorometer (LI-6800-01A, LICOR Inc., Lincoln, NE, USA) with an infrared gas analyzer. Furthermore, the maximum quantum efficiency of PSII photochemistry (F_v_/F_m_) and non-photochemical quenching (NPQ) was obtained in leaves adapted to light and dark (n = 10 replicates per treatment) with a portable fluorimeter FMS-2 (Hansatech Instruments Ltd., King’s Lynn, UK) according to the protocol described by Mateos-Naranjo et al. (2020). Finally, to complete the fluorometry study, delayed fluorescence of leaves of plants subjected to different nitrogen fertilization treatments was detected using a plant imaging system (NightShade LB 985, Berthold Technologies, Germany) equipped with a deeply cooled CCD camera (n = 5 replicates per treatment).

### 2.3 Stable isotopic composition of leaf nitrogen and carbon and nitrogen and carbon concentrations in tissues

The carbon and nitrogen isotopic values of dry and pulverized leaf samples (n = 6 replicates per treatment) were calculated using a Thermo Scientific Delta V spectrometer. Carbon and nitrogen isotope ratios (^13^C/^12^C and ^15^N/^14^N) were expressed in delta (δ) notation, defined as parts per thousand (‰) relative to a standard material (VPDB for δ^13^C and atmospheric N_2_ for δ^15^N). δ^15^N was used as a proxy for nitrogen fluxes, assimilation and allocation (Kalcsits et al., 2014) supplementary to leaf N content. Furthermore, water-use strategies were approached by δ^13^C, which was employed as a proxy for _i_WUE (intrinsic water-use efficiency; Pérez-Harguindeguy et al., 2013) because it relies on the ratio of intercellular to ambient CO_2_ concentrations (C_i_/C_a_) and therefore is affected by transpiration at the stomata (g_s_) and net assimilation (A_N_; Farquhar et al., 1989). Furthermore, the carbon and nitrogen contents of each leaf and root pool replicate (n = 6 replicates per treatment) were quantified using an elemental analyzer (Leco CHNS-932, Madrid, Spain).

### 2.4 Statistical analysis

Statistica software v. 10.0 was used to find variations in plant traits due to experimental treatments using a one-way analysis of variance (ANOVA, test), followed by the *post hoc* LSD test (i.e., Fisher’s least significant difference).

## 3 Results

### 3.1 Strawberry plant development analysis

**Figure 1:**
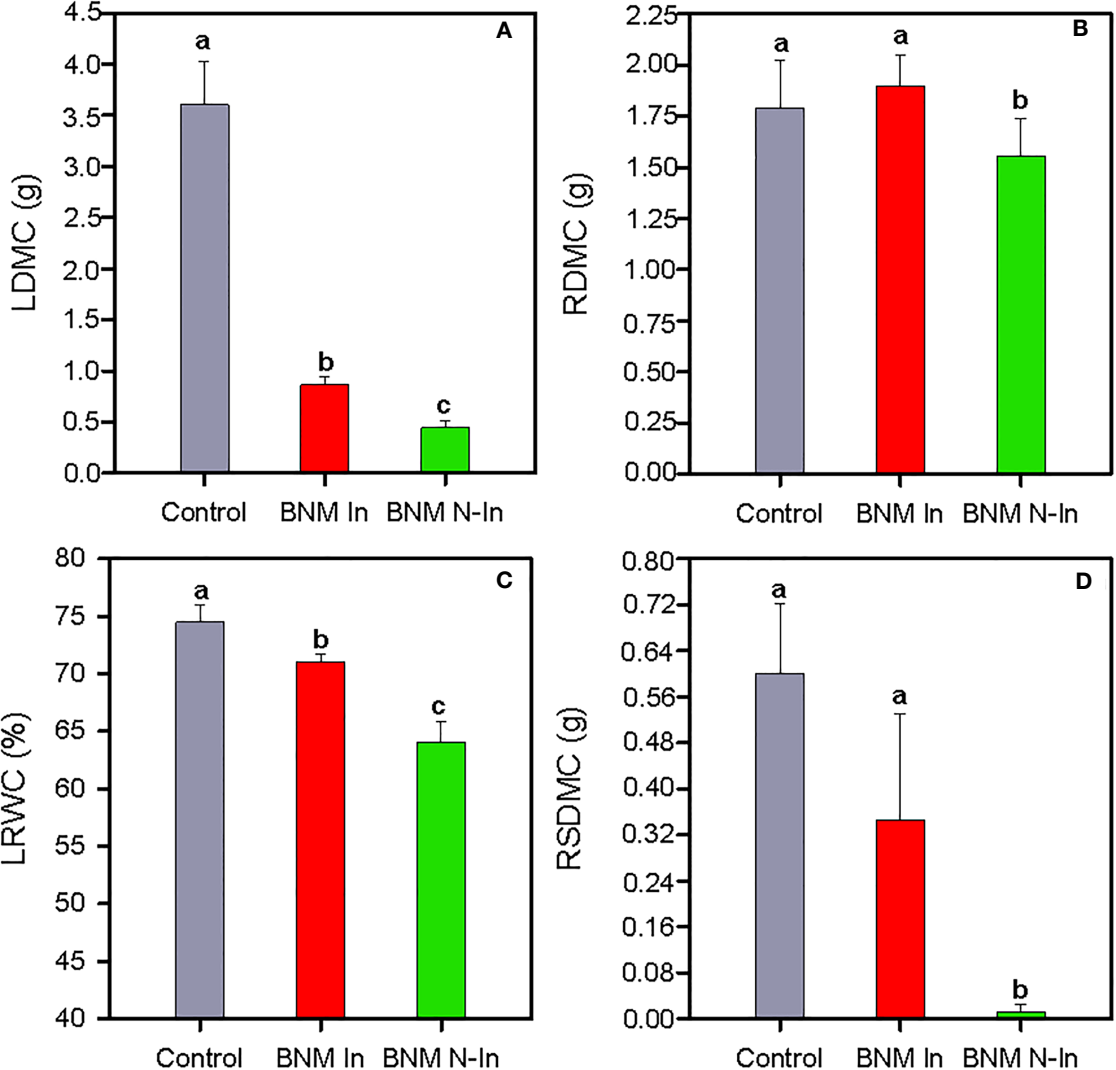
Dry mass content of the leaf, LDMC **(A)**, dry mass content of roots, RDMC **(B)**, relative water content of the leaf, LRWC **(C)** and dry mass content of the reproductive structures, RSDMC **(D)** of strawberry plants subjected to three different nitrogen fertilization treatments after 40 days. Control, chemical fertilization with BNM medium supplemented with soluble nitrogen form; BNM In, chemical fertilization without nitrogen addition and with the inoculation of a designed PGPR-based biofertilizer; and BNM N-In, chemical fertilization without nitrogen addition and inoculation. Values represent mean ± SE of ten replicates. Different letters indicate means that are significantly different from each other (LSD test, p < 0.05).

The limitation of nitrogen fertilization led to a drastic reduction in the growth of strawberry plants after 40 days of treatments. Therefore, compared to the control treatment, the dry mass content of leaves (LDMF) and roots (RDMF) decreased by 88% and 13% in non-inoculated plants grown in the absence of nitrogen (ANOVA, P < 0.05; **Figures 1A, B**). This negative effect was accompanied by a 15% reduction in leaf relative water content (LRWC) (**Figure 1C**), as well as by an almost complete absence of reproductive structures (i.e. flowers and fruits in the initial state of development; **Figure 1D**). However, bacterial inoculation contributed to maintaining root growth at a level similar to control plants and, to some extent, diminished LDMC and LRWC reduction (**Figures 1A–C**). Finally, our results revealed that bacterial inoculation contributed to the presence of reproductive structures, since the values of the dry mass content of reproductive structures (RSDMC) did not significantly differ with respect to control treatment (**Figure 1D**).

### 3.2 Strawberry plant photosynthetic apparatus performance analysis

**Figure 2:**
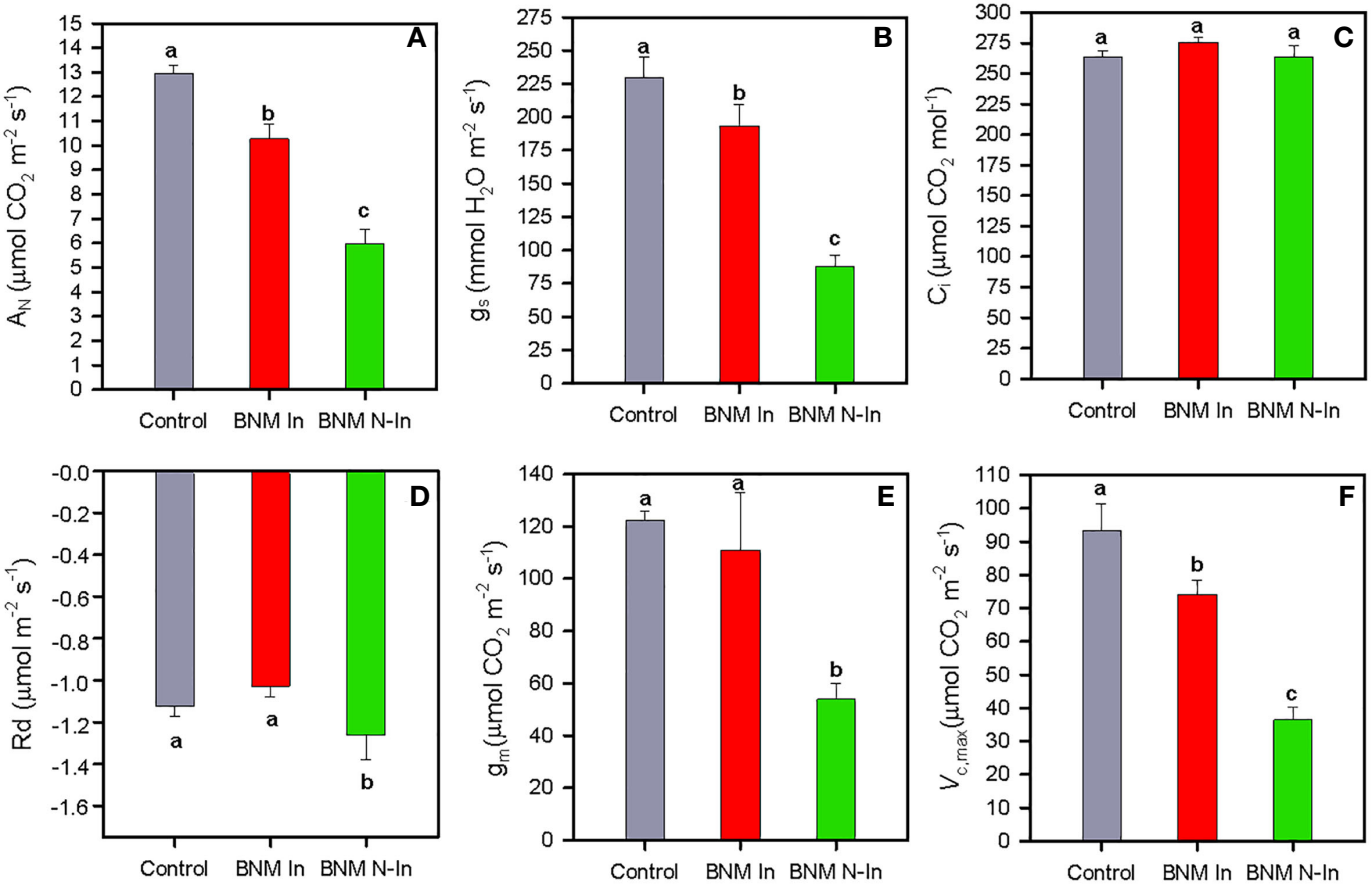
Net photosynthetic rate, A_N_ **(A)**, stomatal conductance, g_s_ **(B)**, intercellular CO_2_ concentration, C_i_ **(C)**, dark respiration, R_d_ **(D)**, mesophyll conductance, g_m_ **(E)** and maximum carboxylation rate allowed by RuBP, *V*_c,max_ **(F)** in radomly selected strawberry leaves of plants subjected to three different nitrogen fertilization treatments and bacterial inoculation after 40 days. For treatment details, see **Figure 1** caption. Values represent mean ± SE of five replicates. Different letters indicate means that are significantly different from each other (LSD test, p < 0.05).

After 40 days of treatment, the net photosynthetic rate (A_N_) showed a sharp decrease in c. 54% in plants grown without nitrogen fertilization compared to their nitrogen-supplied counterparts; however, this harmful effect was buffered by up to 50% due to bacterial inoculation (**Figure 2A**). A very similar pattern was observed for stomatal conductance (g_s_) (ANOVA, P < 0.05; **Figure 2B**), while intercellular CO_2_ concentration (C_i_) showed mean values c. 260 µmol CO_2_ mol^-1^ in all treatments (**Figure 2C**), and dark respiration (R_d_) values were higher in plants grown under N fertilization restriction treatment and without bacterial inoculation (**Figure 2D**).

**Figure 3:**
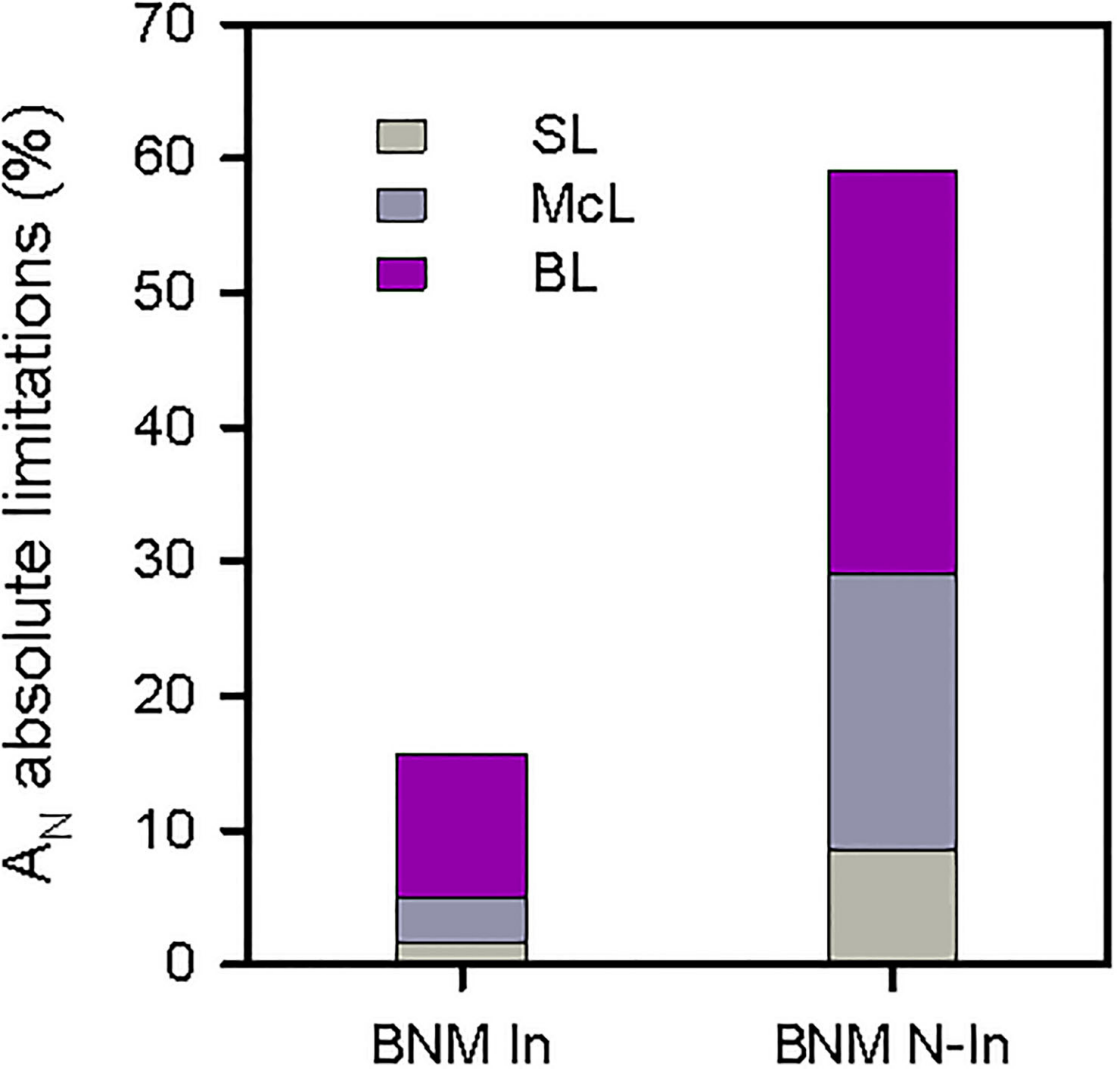
Quantitative analysis of absolute limitations in photosynthesis (A_N_) respect to the control treatment of strawberry plants subjected to nitrogen fertilization restriction with (BNM In) and without bacterial inoculation (BNM N-In) after 40 days. SL, stomatal; McL, mesophyll; BL, biochemical limitations.

On the other hand, our photosynthetic absolute limitation analysis showed that the drastic decrease in A_N_ after 40 days of treatment in non-inoculated plants was mainly due to a biochemical component with a limitation of 30%, followed by limitations in mesophyll conductance (McL) and stomatal (SL) with 20% and 8%, respectively. While in inoculated plants, diffusional (McL+SL) and biochemical restrictions (BL) were 5% and 10%, respectively (**Figure 3**). These response patterns were sustained by the mesophyll conductance (g_m_) and the maximum carboxylation rate allowed by carboxylase/oxygenase of the ribulose-1,5-biphospate (*V*_c,max_) results; therefore, the g_m_ values did not differ significantly between inoculation treatments (**Figure 2E**). And the inhibition of *V*_c,max_ due to the absence of N fertilization was improved to 30% in inoculated plants (ANOVA, P < 0.05; **Figure 2F**).

**Figure 4:**
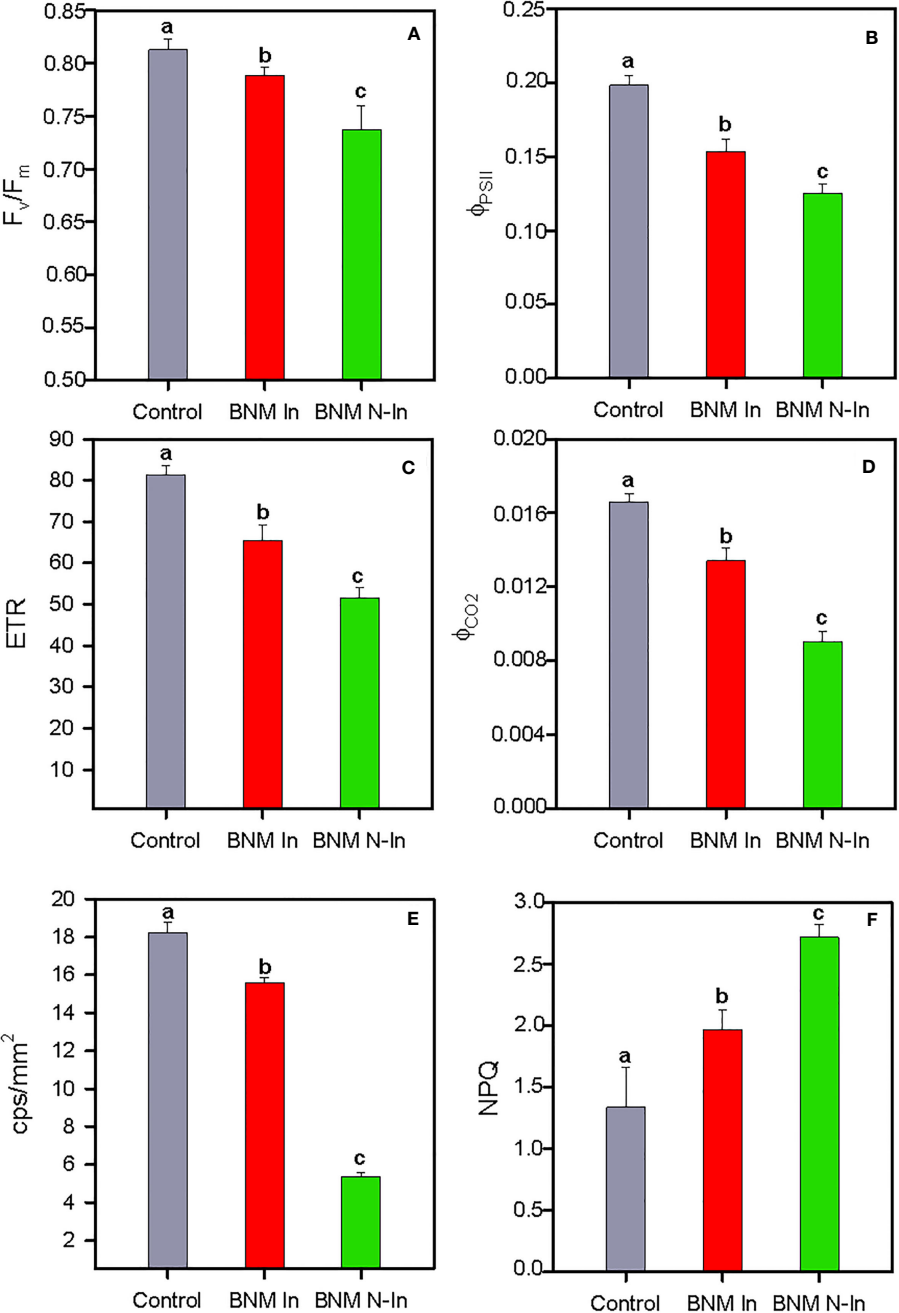
Maximum quantum efficiency of PSII photochemistry, F_v_/F_m_ **(A)**, actual efficiency of the photosystem II, Φ_PSII_ **(B)**, electron transport rate, ETR **(C)**, photosynthesis quantic apparent efficiency, Φ_CO2_ **(D)**, delayed fluorescence in counts per second per surface units, cps/mm^2^ **(F)** and non-photochemical quenching, NPQ **(E)** in radomly selected strawberry leaves of plants subjected to three different nitrogen fertilization treatments after 40 days. For treatment details, see **Figure 1** caption. Values represent mean ± SE of ten replicates for panels **(A–E)**, and five replicates for panel **(F)** Different letters indicate means that are significantly different from each other (LSD test, p < 0.05).

There was a significant effect of nitrogen fertilization limitation and bacterial inoculation on the photochemical traits of the strawberry plant (**Figures 4A–F**). In this sense, overall the maximum quantum efficiency of PSII photochemistry (F_v_/F_m_), actual efficiency of photosystem II (Φ_PSII_), electron transport rate (ETR), and photosynthesis quantic apparent efficiency (Φ_CO2_) values were lower under nitrogen limitation, making this reduction even more acute for non-inoculated plants (ANOVA, P < 0.05; **Figures 4A–D**). A similar pattern was observed for delayed fluorescence in counts per second per surface unit, as indicated by the lowest values recorded for plants fertilized with BNM medium without nitrogen addition and bacterial inoculation (i.e. BNM N-In treatment) (ANOVA, P < 0.05; **Figure 4E**). Furthermore, responses to these parameters were accompanied by an increase in non-photochemical quenching (NPQ), as indicated by the highest values recorded in non-inoculated plants grown without N fertilization (ANOVA, P < 0.05; **Figure 4F**).

### 3.3 Leaf nitrogen and carbon isotopic composition of strawberries and tissue concentration analysis

**Figure 5:**
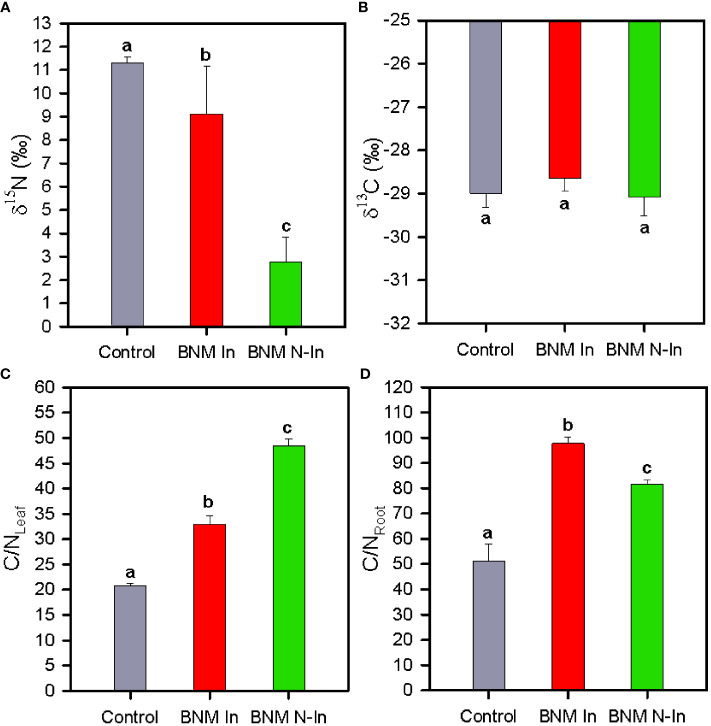
Leaf stable isotope composition δ^15^N **(A)** and δ^13^C **(B)**, leaf C/N ratio **(C)** and root C/N ratio **(D)** in strawberry plants subjected to three different nitrogen fertilization and bacterial inoculation treatments after 40 days. For treatment details, see **Figure 1** caption. Values represent mean ± SE of six replicates. Different letters indicate means that are significantly different from each other (LSD test, p < 0.05).

After 40 days of treatments leaf δ^15^N decreased considerably in plants grown under nitrogen fertilization limitation; being this reduction in high degree offset in inoculated plants (**Figure 5A**). On the contrary, leaf δ^13^C values showed mean values c. -29 parts per thousand in all treatments (**Figure 5B**).

Finally, the C/N ratios of leaves and roots were significantly higher in plants subjected to nitrogen fertilization restriction treatment, although this effect was less pronounced for leaves in inoculated plants with respect to control due to the higher leaf nitrogen content compared to non-inoculated plants (ANOVA, P < 0.05; **Figures 5C, D**). On the contrary, inoculated plants showed the highest C/N concentration of the root due to the higher carbon concentration of the roots relative to the other treatments.

## 4 Discussion

Advances in biotechnology have allowed PGPR to become a promising biotool for crop development in a sustainable manner, due to its multiple positive effects on plants by contributing to the growth of the root system and/or strengthening its tolerance to a number of environmental stresses, including nutritional deficits (Adesemoye et al., 2009; Bhattacharyya & Jha, 2012; Glick, 2012; Khan et al., 2020). According to those studies, we found that the application of the designed PGPR-based biofertilizer had a positive impact on the growth and physiological performance of strawberry plants subjected to a complete nitrogen fertilization restriction scenario, compared to non-inoculated plants. Thus, bacterial inoculation was able to largely reverse the negative impact of nitrogen fertilization withholding on plant growth and fertility traits, as indicated by maintaining the root dry mass content (RDMC) and reproductive structure dry mass content (RSDMC) at a level similar to control treatment and to some extent ameliorated the reduction in leaf dry mass content (LDMC). Our results are also in agreement with previous results found for strawberry, where the presence of microorganisms with PGP properties counterbalanced to some extent the detrimental effects of stressful environmental factors, such as soil salinization, high temperature, and phosphorus deficit, on plant development (Redondo-Gómez et al., 2021; Redondo-Gómez et al., 2022; Valle-Romero et al., 2023).

The positive ameliorative effect of inoculation on strawberry growth under stress induced by N limitation could be explained by some characteristics of the biofertilizer, which would lead to a direct positive effect on plant development, and mainly on its root development. In this sense, several integrant strains of the designed inoculum, particularly strains SRT15 and S110, have the capacity to produce the auxin indole-3-acetic acid (IAA, Valle-Romero et al., 2023), which is a key phytohormone in shaping the architecture of the root and the formation of the lateral root of the plant (Remans et al., 2008; Spolaor et al., 2016). Therefore, the capacity to produce IAA would explain the growth pattern obtained in inoculated strawberry plants compared to their non-inoculated counterparts, as previously described in other horticultural and herb crops (De Silva et al., 2000; Zaidi et al., 2015; Redondo-Gómez et al., 2021; Redondo-Gómez et al., 2022). Similarly, bacterial inoculation would have led to a higher root-to-shoot ratio (data not presented), fact also related to their higher LRWC values. Consequently, their greater root development would have allowed them to explore larger volumes of soil for water acquisition, while, at the same time, they would have lower water losses due to the smaller leaf surface caused by nutritional deficit (Poorter et al., 2011). Furthermore, this pattern of biomass partition fits well with the most common pattern recorded in plants in nutrient-poor environments (Bloom et al., 1985; Bonifas et al., 2005; Mašková and Herben, 2018) with the consequence impact on the rate of plant growth (Poorter et al., 2011). Although, we found that this pattern would be more marked with the inoculation, circumstance that would contribute to partially explain the growth and tolerance differences to nitrogen-induced deficit stress recorded between inoculated and non-inoculated strawberry plants. Similarly, greater availability of water resources in inoculated plants would have made more possible water flows for fruit growth, which would contribute to explain the higher values of RSDMC in these plants, particularly considering that this species is characterized by the development of fruits with a high water content (Grant et al., 2010; Martínez-Ferri et al., 2016).

On the other hand, the ameliorative effect of bacterial inoculation in strawberry development under N limitation conditions was associated with a positive impact on essential steps of photosynthetic metabolism of strawberry compared to non-inoculated plants. Therefore, we found that there were improvements in the carboxylation process and the leaves gas exchange, although without variations in the efficiency of intrinsic water use between plants subjected to different experimental treatments, as indicated the invariable leaf δ^13^C and C_i_ values. Thus, bacterial inoculation was able to buffer up to 50% the reduction in A_N_ due to the lack of nitrogen in the growth medium. This circumstance would have made possible a greater availability of photoassimilates that, together with the aforementioned higher LRWC values, would contribute to explain the beneficial impact of inoculation on strawberry growth and RSDMC values. Specifically, the beneficial ameliorative effect of the inoculation on the carbon assimilation capacity occurred in various essential steps. Thus, our quantitative analysis of absolute photosynthetic limitations indicated that the highest photosynthetic yield in inoculated plants was mediated by a positive effect on photosynthesis diffusion limitations (SL+McL), as indicated by the highest g_s_ and g_m_ values. Additionally, bacterial inoculation led to a reduction in biochemical limitations, but to a lesser extent, as shown in the higher values of the maximum carboxylation rate allowed by carboxylase/oxygenase of ribulose-1,5-biphospate (*V*_c,max_) compared to non-inoculated plants, as previously described in strawberry in response to salt stress and phosphorus deficit (Selvi et al., 2020; Redondo-Gómez et al., 2021; Valle-Romero et al., 2023). Similarly, the positive effect was attributed partially to an improvement in leaf light harvesting efficiency, together with adequate coordination with its carboxylation phase, compared with non-inoculated plants. In this way, we found that N-deficit caused negative effects on PSII functionality of strawberry plants, as indicated by the lower F_v_/F_m_ and Φ_PSII_ values, in agreement with previous studies (Morales and Warren, 2012). However, bacterial inoculation countered this reduction to a large extent. These results, together with the higher delayed fluorescence recorded, would indicate a greater integrity of the chloroplast thylakoid membrane of inoculated strawberry plants compared with their non-inoculated counterparts (Strasser et al., 2004). Furthermore, there was a positive effect on the functioning of the electron transport chain according to the highest ETR values. This circumstance highlights the role of inoculation in maintaining homeostasis between captured energy and plant carboxylation capacity, this idea being also supported by the highest photosynthesis quantic apparent efficiency (Φ_CO2_) values recorded in these plants. On the contrary, in non-inoculated plants, nitrogen deficit conditions would lead to an increase in NPQ and R_d_ values, indicating that much absorbed energy would not have taken the photochemical pathway (Flexas et al., 2013), with a consequent negative impact on photoassimilates availability for strawberry plant development and fruit production.

Interestingly, our results revealed that the improved effect of bacterial inoculation on strawberry photosynthetic metabolisms under N limitation conditions was linked to a better nutritional balance of the plant. In this sense, we should highlight that nitrogen is an essential element for the photosynthetic process (Evans and Clarke, 2019). In fact, several authors have found a positive relation between leaf N concentration or N fertilization practices and photosynthetic apparatus performance, in terms of improving plant carboxylation, photochemical, and CO_2_ diffusion capacity (Warren, 2004; Liao et al., 2019). Consistent with these results, we found a lower leaf C/N ratio in inoculated plants, which was associated with a higher nitrogen content in their leaves and, therefore, could be explained by the fixation capacity of N of strains SMT38, SRT15, and S110. This effect was also supported by stable leaf nitrogen isotopic analysis. Therefore, the inoculated plants showed leaf δ^15^N values close to control plants compared to their non-inoculated counterparts. However, contrary to what was expected, both treatments showed δ^15^N values higher (11.3 and 9.1‰ respectively) than non-inoculated plants (2.8‰), despite the fact that we expected isotopic signals close to cero related to the bacterial biological fixation process (Kalcsits et al., 2014). This unexpected result could be explained by the inoculation process itself. Thus, when analyzing the entire plant for nitrogen balance, other processes are involved, including death and turnover of the microorganism (Unkovich, 2013). In this sense, a study by Dijkstra et al. (2006) proposed that the increase of δ^15^N of the soil organic matter could be due to microbial biomass itself. However, despite this result, both the concentration of leaf nitrogen tissues and the stable isotopic analysis would suggest that bacterial inoculation would have led to an increased nitrogen source available in the medium, favoring the nutritional balance of strawberry, and consequently, its photosynthetic metabolism and development, as was recorded in this study. Nevertheless, it should be noted that bacterial inoculation was unable to fully cover the nutritional needs of the plants by itself, since plant development did not reach the levels registered in the control plants. This circumstance points towards a combined strategy to improve nitrogen fertilization practices efficiency in strawberry cultivation, which includes the use of PGPR-based biofertilizer in combination with the limited application of chemical nitrogen fertilizer. Therefore, this area deserves further future research.

## 5 Conclusions

The PGPR-based biofertilizer designed composed by self-compatible stress tolerance strains isolated from halophytic plant species was unable to completely replace the complete withdrawal of nitrogen chemical fertilization in strawberry crop. However, it was able to significantly improve *Fragaria x ananassa* cv. *Rociera* tolerance to nutritionally induced stress. Therefore, although nitrogen limitation decreased considerable plant areal biomass development, this effect was mitigated to a certain extent by the inoculated bacteria. Furthermore, inoculated plants were able to maintain root growth and fertility traits development at a level similar to that of those plants supplied with an adequate level of nitrogen chemical fertilization.

The positive effect of the PGPR-based biofertilizer on plant growth was mediated by an ameliorative effect of the negative impact of the N deficit on strawberry photosynthetic metabolism in terms of reduced carbon diffusional and biochemical limitations, while the efficiency of PSII photochemistry was maintained in terms of light-harvesting efficiency, together with adequate coordination with the carboxylation phase. Furthermore, inoculation improved nitrogen nutritional balance, which was also associated with a beneficial impact on plant photosynthetic metabolism. Therefore, these circumstances make this biofertilizer a promising complementary tool for improving the nitrogen-based chemical fertilization process with nitrogen in strawberry farms, allowing a substantial reduction in the application of this agrochemical.

## Data availability statement

The raw data supporting the conclusions of this article will be made available by the authors, without undue reservation.

## Author contributions

EM-N and. SR-G conceived the study, supervised, and acquired funding for the project; EM-N gathered the data, designed and performed the analyses and wrote the manuscript with the help of JG-L, MZ and SR-G; NF-D with the assistance of IR-L and EP, provided the bacterial inocula. All authors performed the experimental development, provided corrections to manuscript drafts, and discussed ideas within it. All authors have read and agreed to the published version of the manuscript.

## References

[B1] Adesemoye A. O. Torbert H. A. Kloepper J. W. (2009). Plant growth-promoting rhizobacteria allow reduced application rates of chemical fertilizers. Microb. Ecol. 58, 921–929. doi: 10.1007/s00248-009-9531-y 19466478

[B2] Andrades-Moreno L. Del Castillo I. Parra R. Doukkali B. Redondo-Gómez S. Pérez-Palacios P.. (2014). Prospecting metal-resistant plant-growth promoting rhizobacteria for rhizoremediation of metal contaminated estuaries using *Spartina densiflora.* Environ. Sci. pollut. Res. 21, 3713–3721. doi: 10.1007/s11356-013-2364-8 24281681

[B3] Ariza M. T. Soria C. Medina-Mínguez J. J. Martínez-Ferri E. (2012). Incidence of misshapen fruits in strawberry plants grown under tunnels is affected by cultivar, planting date, pollination, and low temperature. HortScience 47, 1569–1573. doi: 10.21273/HORTSCI.47.11.1569

[B4] Arregui L. M. Quemada M. (2006). Drainage and nitrate leaching in a crop rotation under different N-fertilizer strategies: application of capacitance probes. Plant Soil 288, 57–69. doi: 10.1007/s11104-006-9064-9

[B5] Balasooriya H. N. Dassanayake K. B. Seneweera S. Ajlouni S. (2018). Interaction of elevated carbon dioxide and temperature on strawberry (*Fragaria × ananassa*) growth and fruit yield. Int. J. Agric. Biosyst. Eng. 12, 279–287.

[B6] Barros R. Isidoro D. Aragüés R. (2012). Irrigation management, nitrogen fertilization and nitrogen losses in the return flows of La Violada irrigation district (Spain). Agric. Ecos. Environ. 155, 161–171. doi: 10.1016/j.agee.2012.04.004

[B7] Bhattacharyya P. N. Jha D. K. (2012). Plant growth-promoting rhizobacteria (PGPR): Emergence in agriculture. World J. Microbiol. Biotech. 28, 1327–1350. doi: 10.1007/s11274-011-0979-9 22805914

[B8] Billen G. Garnier J. Lassaletta L. (2013). The nitrogen cascade from agricultural soils to the sea: Modelling nitrogen transfers at regional watershed and global scales. Philos. Trans. R. Soc B Biol. Sci. 368, 20130123. doi: 10.1098/rstb.2013.0123 PMC3682743 23713121

[B9] Bloom A. J. Chapin F. S. III Mooney H. A. (1985). Resource limitation in plants–an economic analogy. Ann. Rev. Ecol. Evol.Syst. 16, 363–392. doi: 10.1146/annurev.es.16.110185.002051

[B10] Bonifas K. D. Walters D. T. Cassman K. G. Lindquist J. L. (2005). Nitrogen supply affects root:shoot ratio in corn and velvetleaf (*Abutilon theophrasti*). Weed Sci. 53, 670–675. doi: 10.1614/WS-05-002R.1

[B11] Bottoms T. G. Hartz T. K. Cahn M. D. Farrara B. F. (2013). Crop and soil nitrogen dynamics in annual strawberry production in California. HortScience horts. 48, 1034–1039. doi: 10.21273/HORTSCI.48.8.1034

[B12] Davis K. F. Gephart J. A. Emery K. A. Leach A. M. Galloway J. N. (2016). Meeting future food demand with current agricultural resources. Glob. Environ. Change 39, 125–132. doi: 10.1016/j.gloenvcha.2016.05.004

[B13] De Silva A. Patterson K. Rothrock C. Moore J. (2000). Growth promotion of highbush blueberry by fungal and bacterial inoculants. Hortscience 35, 1228–1230. doi: 10.21273/HORTSCI.35.7.1228

[B14] Dijkstra P. Ishizu A. Doucett R. Hart S. C. Schwartz E. Menyailo O. V.. (2006). C-13 and N-15 natural abundance of the soil microbial biomass. Soil Biol. Biochem. 38, 3257–3266. doi: 10.1016/j.soilbio.2006.04.005

[B15] Ehrhardt D. W. Atkinson E. M. Long S. R. (1992). Depolarization of alfalfa root hair membrane potential by Rhizobium meliloti Nod factors. Science 256 998–1000. 10744524 10.1126/science.10744524

[B16] Etesami H. Maheshwari D. K. (2018). Use of plant growth promoting rhizobacteria (PGPRs) with multiple plant growth promoting traits in stress agriculture: Action mechanisms and future prospects. Ecotox. Environ. Safe 156, 225–246. doi: 10.1016/j.ecoenv.2018.03.013 29554608

[B17] Evans J. R. Clarke V. C. (2019). The nitrogen cost of photosynthesis. J. Exp. Bot. 70, 7–15. doi: 10.1093/jxb/ery366 30357381

[B18] Falkenmark M. Rockstrom J. (2004). Balancing water for humans and nature: the new approach to ecohydrology. London: Earthscan.

[B19] FAO Food and Agricultural Organization. Available at: http://www.fao.org (Accessed 1 June 2023).

[B20] Farquhar G. D. Ehleringer J. R. Hubick K. T. (1989). Carbon isotope discrimination and photosynthesis. Ann. Rev. Plant Physiol. Plant Mol. Biol. 40, 503. doi: 10.1146/annurev.pp.40.060189.002443

[B21] Flexas J. Baron M. Bota J. (2009). Photosynthesis limitations during water stress acclimation and recovery in the drought-adapted Vitis hybrid Richter-110 (*V. berlandieri x V. rupestris*). J. Exp. Bot. 60, 2361–2377. doi: 10.1093/jxb/erp069 19351904

[B22] Flexas J. Niinemets Ü. Galle A. Barbour M. M. Centritto M. Diaz-Espejo A.. (2013). Diffusional conductances to CO_2_ as a target for increasing photosynthesis and photosynthetic water-use efficiency. Photosynth. Res. 117, 45–59. doi: 10.1007/s11120-013-9844-z 23670217

[B23] Flores-Duarte N. J. Pérez-Pérez J. Navarro-Torre S. Mateos-Naranjo E. Redondo-Gómez S. Pajuelo E.. (2022). Improved *Medicago sativa* nodulation under stress assisted by *Variovorax* sp. endophytes. Plants 11, 1091. doi: 10.3390/plants11081091 35448819 PMC9026315

[B24] Foley J. A. Ramankutty N. Brauman K. A. Cassidy E. S. Gerber J. S.. (2011). Solutions for a cultivated planet. Nature 478, 337–342. doi: 10.1038/nature10452 21993620

[B25] Glick B. R. (2012). Plant growth-promoting bacteria: Mechanisms and applications. Scientifica 2012, 963401. doi: 10.6064/2012/963401 24278762 PMC3820493

[B26] Grant O. M. Johnson A. W. Davies M. J. James C. M. Simpson D. W. (2010). Physiological and morphological diversity of cultivated strawberry (*Fragaria×ananassa*) in response to water deficit. Environ. Exp. Bot. 68, 264–272. doi: 10.1016/j.envexpbot.2010.01.008

[B27] Grassi G. Magnani F. (2005). Stomatal, mesophyll conductance and biochemical limitations to photosynthesis as affected by drought and leaf ontogeny in ash and oak trees. Plant Cell Environ. 28, 834–849. doi: 10.1111/j.1365-3040.2005.01333.x

[B28] Hartmann T. E. Yue S. C. Schulz R. He X. K. Chen X. P. Zhang F. S.. (2015). Yield and n use efficiency of a maize–wheat cropping system as affected by different fertilizer management strategies in a farmer’s field of the north China plain. Field Crops Res. 174, 30–39. doi: 10.1016/j.fcr.2015.01.006

[B29] Kalcsits L. A. Buschhaus H. A. Guy R. D. (2014). Nitrogen isotope discriminationas an integrated measure of nitrogenfluxes, assimilation and allocation inplants. Physiol. Plantarum 151, 293–304. doi: 10.1111/ppl.12167 24512444

[B30] Khan A. Ding Z. T. Ishap M. Khan I. Ahmed A. A. Khan A. Q.. (2020). Applications of beneficial plant growth promoting rhizobacteria and mycorrhizae in rhizosphere and plant growth: A review. Int. J. Agric. Biol. Eng. 13, 199–208. doi: 10.25165/j.ijabe.20201305.5762

[B31] Lhada J. K. Pathak H. Krupnik T. J. Six J. Kessel C. (2005). Efficiency of fertilizer nitrogen in cereal production: retrospects and prospects. Adv. Agron. 87, 85–156. doi: 10.1016/S0065-2113(05)87003-8

[B32] Liao L. Dong T. Qiu X. Rong Y. Wang Z. Zhu J. (2019). Nitrogen nutrition is a key modulator of the sugar and organic acid content in citrus fruit. Plots One 14, e0223356. doi: 10.1371/journal.pone.0223356 PMC6786551 31600253

[B33] Ljung K. (2013). Auxin metabolism and homeostasis during plant development. Development 140, 943–950. doi: 10.1242/dev.086363 23404103

[B34] Long S. P. Bernacchi C. J. (2003). Gas exchange measurements, what can they tell us about the underlying limitations to photosynthesis? Procedures and sources of error. J. Exp. Bot. 54, 2393–2401. doi: 10.1093/jxb/erg262 14512377

[B35] Lozano D. Ruiz N. Gavilán P. (2016). Consumptive water use and irrigation performance of strawberries. Agric. Water Manage. 169, 44–51. doi: 10.1016/j.agwat.2016.02.011

[B36] Marinov I. Marinov A. M. (2014). A coupled mathematical model to predict the influence of nitrogen fertilization on crop, soil and groundwater quality. Water Resour. Manage 28, 5231–5246. doi: 10.1007/s11269-014-0664-5

[B37] Martinez-Dalmau J. Berbel J. Ordóñez-Fernández R. (2021). Nitrogen fertilization. A review of the risks associated with the inefficiency of its use and policy responses. Sustainability 13, 5625. doi: 10.3390/su13105625

[B38] Martínez-Ferri E. Soria C. Ariza M. T. Medina J. J. MIranda L. Domínguez P.. (2016). Water relations, growth and physiological response of seven strawberry cultivars (*Fragaria × ananassa Duch.*) to different water availability. Agric. Water Mang. 164, 73–82. doi: 10.1016/j.agwat.2015.08.014

[B39] Mašková T. Herben T. (2018). Root:shoot ratio in developing seedlings: How seedlings change their allocation in response to seed mass and ambient nutrient supply. Ecol. Evol. 8, 7143–7150. doi: 10.1002/ece3.4238 30073073 PMC6065327

[B40] Mateos-Naranjo E. López-Jurado J. Redondo-Gómez S. Pérez-Romero S. Glick B. R. Rodríguez-Llorente I. D.. (2020). Uncovering PGPB *Vibrio spartinae* inoculation-triggered physiological mechanisms involved in the tolerance of *Halimione portulacoides* to NaCl excess. Plant Physiol. Biochem. 154, 151–159. doi: 10.1016/j.plaphy.2020.05.034 32559519

[B41] Mekonnen M. M. Hoekstra A. Y. (2014). Water footprint benchmarks for crop production: A first global assessment. Ecol. Indic. 46 214–223. doi: 10.1016/j.ecolind.2014.06.013

[B42] Mesa J. Mateos-Naranjo E. Caviedes M. A. Redondo-Gómez S. Pajuelo E. Rodríguez-Llorente I. D. (2015). Scouting contaminated estuaries: Heavy metals resistant and plant growth promoting rhizobacteria in the native metal rhizoaccumulator *Spartina maritima* . Mar. pollut. Bull. 90, 150–159. doi: 10.1016/j.marpolbul.2014.11.002 25467875

[B43] Mesa-Marín J. Pérez-Romero J. A. Redondo-Gómez S. Pajuelo E. Rodríguez-Llorente I. D. Mateos-Naranjo E. (2020). Impact of plant growth promoting bacteria on *Salicornia ramosissima* ecophysiology and heavy metal phytoremediation capacity in estuarine soils. Front. Microbiol. 17, 553018. doi: 10.3389/fmicb.2020.553018 PMC7527472 33042058

[B44] Morales F. Warren C. R. (2012). “Photosynthesis Responses to Nutrient Deprivation and Toxicities,” in Terrestrial Photosynthesis in a Changing Environment. Eds. Flexas J. Loreto F. Medrano I. (Cambridge, UK: Cambridge University Press), 312–330.

[B45] Navarro-Torre S. Mateos-Naranjo E. Caviedes M. A. Pajuelo E. Rodríguez-Llorente I. D. (2016). Isolation of plant-growth-promoting and metal-resistant cultivable bacteria from Arthrocnemum macrostachyum in the Odiel marshes with potential use in phytoremediation. Mar. pollut. Bull. 110, 133–142. doi: 10.1016/j.marpolbul.2016.06.070 27349383

[B46] Pérez-Harguindeguy N. Díaz S. Garnier E. Lavorel S. Poorter H. Jaureguiberry P.. (2013). New handbook for standardised measurement of plant functional traits worldwide. Austral. J. Bot. 61, 167–234. doi: 10.1071/BT12225

[B47] Pons T. L. Flexas J. von Caemmerer S. Evans J. R. Genty B. Ribas-Carbo M.. (2009). Estimating mesophyll conductance to CO_2_: Methodology, potential errors, and recommendations. J. Exp. Bot. 60, 2217–2234. doi: 10.1093/jxb/erp081 19357431

[B48] Poorter H. Niklas K. J. Reich P. B. Oleksyn J. Poot P. Mommer L. (2011). Biomass allocation to leaves, stems and roots:meta-analyses of interspecific variation andenvironmental control. New Phytol. 193, 30–50. doi: 10.1111/j.1469-8137.2011.03952.x 22085245

[B49] Redondo-Gómez S. Mesa-Marín J. Pérez-Romero J. A. López-Jurado J. García-López J. V. Mariscal V.. (2021). Consortia of plant-growth-promoting rhizobacteria isolated from halophytes improve response of eight crops to soil salinization and climate change conditions. Agronomy 11, 1609. doi: 10.3390/agronomy11081609

[B50] Redondo-Gómez S. ROmano-Rodríguez E. Mesa-Marín J. Sola-Elías C. Mateos-Naranjo E. (2022). Consortia of plant-growthpromoting rhizobacteria isolated from halophytes improve the response of swiss chard to soil salinization. Agronomy 12, 468. doi: 10.3390/agronomy12020468

[B51] Remans R. Beebe S. Blair M. Manrique G. Tovar E. Rao I.. (2008). Physiological and genetic analysis of root responsiveness to auxin-producing plant growth-promoting bacteria in common bean (*Phaseolus vulgaris* L.). Plant Soil 302, 149–161. doi: 10.1007/s11104-007-9462-7

[B52] Ruzzi M. Aroca R. (2015). Plant growth-promoting rhizobacteria act as biostimulants in horticulture. Sci. Horticul. 196, 124–134. doi: 10.1016/j.scienta.2015.08.042

[B53] Selvi T. Eşitgen A. Bayramoğlu Z. Dönmez M. F. (2020). The effect of bacterial applications on resource utilization ın strawberry (*Fragaria x ananassa duch*) production. KSU J. Agric. Nat. 23, 1308–1313. doi: 10.18016/ksutarimdoga.vi.631957

[B54] Spolaor L. T. Goncalves L. S. A. dos Santos O. J. A. P. de Oliveira A. L. M. Scapim C. A. Bertagna F. A. B.. (2016). Plant growth-promoting bacteria associated with nitrogen fertilization at topdressing in popcorn agronomic performance. Bragantia 75, 33–40. doi: 10.1590/1678-4499.330

[B55] Strasser R. J. Tsimilli-Michael M. Srivastava A. (2004). “Analysis of the chlorophyll a fluorescence transient,” in Chlorophyll a Fluorescence: A Signature of Photosynthesis. Eds. Papageorgiou G., Govindjee C. (Dordrecht: Springer Netherlands), 321–362.

[B56] Tilman D. Balzer C. Hill J. Befort B. L. (2011). Global food demand and the sustainable intensification of agriculture. Proc. Natl. Acad. Sci. U.S.A. 108, 20260–20264. doi: 10.1073/pnas.1116437108 22106295 PMC3250154

[B57] Unkovich M. (2013). Isotope discrimination provides new insight into biological nitrogen fixation. New Phytol. 198, 643–646. doi: 10.1111/nph.12227 23461709

[B58] Valle-Romero P. García-López J. V. Redondo-Gómez S. Flores-Duarte N. J. Rodríguez-Llorente I. D. Idaszkin Y. L.. (2023). Biofertilization with PGP bacteria improve strawberry plant performance under sub-optimum phosphorus fertilization. Agronomy 13, 335. doi: 10.3390/agronomy13020335

[B59] Warren C. R. (2004). The photosynthetic limitation posed by internal conductance to CO_2_ movement is increased by nutrient supply. J. Exp. Bot. 55, 2313–2321. doi: 10.1093/jxb/erh239 15310814

[B60] Xu H. Liu M. Tang Y. Zhao F. Cao W. He M.. (2023). Optimized management strategy increased grain, yield, promoted nitrogen balance, and improved water productivity in winter wheat. Front. Plant Sci. 14, 1182568. doi: 10.3389/fpls.2023.1182568 37324712 PMC10267738

[B61] Yadav S. K. Khokhar U. U. Sharma S. D. Kumar P. (2016). Response of strawberry to organic versus inorganic fertilizers. J. Plant Nutr. 39, 194–203. doi: 10.1080/01904167.2015.1109115

[B62] Zaidi A. Ahmad E. Khan M. S. Saif S. Rizvi A. (2015). Role of plant growth promoting rhizobacteria in sustainable production of vegetables: Current perspective. Sci. Horticul. 193, 231–239. doi: 10.1016/j.scienta.2015.07.020

